# A call-to-action: integrate a learning health system framework into longitudinal population studies to improve health response in Africa

**DOI:** 10.1093/haschl/qxae010

**Published:** 2024-01-31

**Authors:** Damazo T Kadengye, Agnes N Kiragga

**Affiliations:** Data Science Program, African Population and Health Research Center (APHRC), P.O. Box 10787-00100, Nairobi, Kenya; Department of Economics and Statistics, Kabale University, P.O. Box 317, Kabale, Uganda; Data Science Program, African Population and Health Research Center (APHRC), P.O. Box 10787-00100, Nairobi, Kenya

**Keywords:** longitudinal population studies, learning health system, health and demographic surveillance systems

## Abstract

Longitudinal population studies (LPSs) in Africa have the potential to become powerful engines of change by adopting a learning health system (LHS) framework. This is a call-to-action opinion and highlights the importance of integrating an LHS approach into LPSs, emphasizing their transformative potential to improve population health response, drive evidence-based decision making, and enhance community well-being. Operators of LPS platforms, community members, government officials, and funding agencies have a role to contribute to this transformative journey of driving evidence-based interventions, promoting health equity, and fostering long-term public health solutions for African communities.

## The data revolution and population health response in Africa

African countries continue to work towards achieving commitments such as the 2030 Sustainable Development Goals (SDGs)^1^ and the 2063 African Union Agenda.^2^ During the SDGs era, monitoring progress towards achievement of these global and continental development agendas requires availability of timely and quality data at both the national and subnational levels. Therefore, by embracing an aspirational and ambitious Data Revolution,^3^ African countries have a more realistic chance of tracking progress towards these agendas and influencing the trajectories of respective countries in a timely and sustainable manner. The 2015 Africa Data Consensus emphasizes the need for timely and quality data to inform policy development and implementation for development on the continent.^4^ It envisions that each component of the data systems is steered towards establishing a conducive environment for leveraging data, establishing appropriate data infrastructure, while building on and strengthening local capacities for collecting and using data to ensure a holistic response to development priorities. The 2016 Africa Data Revolution report highlights a profound shift in the way that data are being harnessed to impact development decision making, with a particular emphasis on building a culture of usage.^5^ Therefore, leveraging a wide range of data, to review progress against set targets on health and well-being, is critical in improving the effectiveness and sustainability of health systems.

## The role of longitudinal population studies

Longitudinal population studies (LPSs), also known as Health and Demographic Surveillance Systems, enable the collection and processing of data on core components of demographic change and other population- and health-related issues within well-delineated areas. Whereas the primary focus of LPS sites is collection of civil registration and vital statistics (CRVS), they also provide platforms for specialized studies to help understand drivers of population health inequities among the community members.^6-9^ Therefore, LPS platforms are invaluable tools for monitoring population health and informing public health interventions in Africa. In order to contribute to the Data Revolution in Africa, institutions operating LPS platforms need to work closely together to enhance data discoverability and promote responsible open data sharing to inform evidence-informed decision making and policy in Africa. This can be realized through the creation of communities of practice whose objective is to enhance population-based health data sharing, standardization, and harmonization in order to provide reliable data that adhere to the principles of Findability, Accessibility, Interoperability, and Reusability (FAIR).^10^ Some efforts to this end have been established in Africa, such as the Implementation Network for Sharing Population Information from Research Entities (INSPIRE) in East Africa,^11^ the International Network for the Demographic Evaluation of Populations and Their Health (INDEPTH) in Western Africa,^12^ and the South African Population Research Infrastructure Network (SAPRIN) in South Africa.^13^ By ensuring FAIR data through communities of practice of LPS platforms, nongovernmental organizations (NGOs) and institutions operating LPSs have a potential to advance public health response through the creation of tools for knowledge access co-designed with end-users—both communities and policy actors –in order to respond to important policy questions at local, national, and regional levels.

## The learning health system framework

Ensuring FAIR data and benchmarking communities of practice alone will not drive health systems improvement, and some challenges will remain. For most countries, effective sustainable health care improvement appears to be an intractable problem, and there is a recognized vital need for systems-level change to improve health care using an iterative learning health system (LHS) approach.^14^ The LHS approach encompasses integration of research, clinical practice, and continuous learning through operationalization and conversion of routinely collected health data into useful information that enables informed, timely decisions to optimize health care delivery and population health outcomes.^14-17^ As has been documented elsewhere, LHSs are where “science, informatics, incentives, and culture are aligned for continuous improvement and innovation, with best practices seamlessly embedded in the delivery process and new knowledge captured as an integral by-product of the delivery experience.”^15,18^ While the concept of LHS has increasingly generated enthusiasm across public health sectors across the world, its implementation in LPSs in Africa is unknown, to the best of our knowledge. Furthermore, whereas it has been well documented that LPSs are invaluable tools for monitoring population health and informing public health interventions in Africa, there is no known universal consensus among NGOs/institutions operating LPSs, communities, and policy actors around how they contribute to public health improvement at scale. Therefore, there is a need to clarify how NGOs/institutions can integrate the LHS concept into the LPSs they operate.

## Ways for integrating the LHS framework in LPS platforms

By embracing the LHS approach, NGOs/institutions operating LPS platforms can leverage their existing data-collection infrastructure and expand their capabilities to become dynamic engines for generating knowledge and fostering evidence-based policies and interventions through a number of areas, as discussed in the following sections.

### Data integration, analysis, and evidence generation

Incorporating the principles of an LHS, NGOs/institutions operating LPS platforms can integrate diverse data sources beyond CRVS, such as electronic health records, clinical data, environmental data, and socioeconomic indicators. By combining these datasets, LPSs can gain a comprehensive understanding of health trends, risk factors, and the social determinants of health. Advanced data analytics techniques, including data mining, machine learning, and artificial intelligence, can extract actionable insights, predict health outcomes, and guide effective decision making.

### Continuous learning and research

Central to the LHS framework is the promotion of continuous learning and research within LPS communities. The wealth of data collected by NGOs/institutions operating LPS platforms can serve as a valuable resource for research studies, epidemiological investigations, and program evaluations. Collaboration between LPS platforms, research institutions, and local stakeholders facilitates knowledge generation, ensuring that interventions are contextually relevant and evidence-based. Continuous learning enables the translation of research findings into improved policies and practices, leading to better population health outcomes.

### Feedback loop for quality improvement

An integral part of the LHS framework is the establishment of feedback loops to enable continuous improvement. Nongovernmental organizations/institutions operating LPS platforms should actively engage with health care providers, policymakers, and communities to gather feedback on interventions, programs, and health service delivery. This feedback informs quality-improvement initiatives and facilitates the refinement and adaptation of interventions based on real-time data and community needs. Continuous quality improvement drives the effectiveness and efficiency of public health responses.

### Community engagement and empowerment

Community engagement is a cornerstone of an LHS framework. Nongovernmental organizations/institutions operating LPS platforms should actively involve community members in data collection, research initiatives, and decision-making processes by intentionally packaging LPS data and evidence in formats that can easily be understood by local communities. By valuing community perspectives, LPSs foster trust, empower individuals, and promote health ownership. Engaged communities become active participants in designing and implementing interventions, ensuring their relevance, acceptability, and effectiveness. Community engagement enhances the sustainability and impact of public health interventions.

### Partnerships and collaborations

Successful implementation of an LHS in LPSs requires partnerships and collaborations among various stakeholders. Nongovernmental organizations/institutions operating LPS platforms can establish collaborations with governmental agencies, NGOs, academic institutions, and health care providers. These partnerships facilitate the exchange of knowledge, resources, and best practices, driving the adoption of evidence-based interventions and policies. Collaborative efforts amplify the impact of research and support the implementation of effective interventions that address the specific health needs of the communities served by LPSs.

### Ethical considerations and data governance

As NGOs/institutions operating LPS platforms expand their data-collection and -sharing efforts, ensuring robust ethical considerations and data governance is essential. Safeguarding individual privacy, obtaining informed consent, and protecting data security are paramount. Transparent data-governance frameworks, including anonymization techniques and secure storage, foster trust among community members and stakeholders. Upholding ethical practices and transparent data-governance principles contributes to the sustainability, credibility, and ethical use of LPS data.

## A call to action

Integrating an LHS framework into LPS platforms holds immense promise for advancing public health in Africa. By embracing data integration and analysis, promoting continuous learning and research, engaging communities, fostering partnerships, and upholding ethical considerations, NGOs/institutions operating LPSs can optimize their impact on population health. The LHS approach empowers LPS platforms to generate actionable insights, promote evidence-based decision making, and drive sustainable improvements in public health outcomes. Embracing this framework is a transformative step towards creating healthier communities and achieving equitable and sustainable development in Africa.

### To operators of LPS platforms

We urge NGOs operating LPS sites in Africa to embrace the LHS framework and collaborate with LPS platforms to support data integration, research initiatives, and continuous learning. By leveraging resources and expertise, together we can drive evidence-based interventions, improve public health outcomes, and foster sustainable development in the communities we serve.

### To communities under LPS

We urge NGOs/institutions operating LPS sites to actively engage community members within the demographic surveillance areas of LPS platforms to share insights that contribute to data-collection efforts and demand for use of evidence generated from the data to address the needs or gaps specific to their communities. By getting involved in research initiatives and decision-making processes, community members can shape interventions that directly address their health needs.

### To subnational and national governments

We call upon the government to recognize the potential of LPS platforms as key drivers of improved public health in Africa. We urge African governments to provide direct financial and resource support to NGOs/institutions operating LPS platforms in their countries to ensure country ownership and sustainability of LPS platforms. Governments should invest in the integration of an LHS framework into LPS operations, providing necessary resources and support. By prioritizing evidence-based decision making, fostering partnerships, and upholding ethical considerations, local and national health care systems can be strengthened, thereby reducing health disparities and achieving better health outcomes for all citizens.

### To funding agencies

Last, to funding agencies supporting health initiatives in Africa, we emphasize the importance of investing in the advancement of LPS platforms towards the LHS framework. By allocating resources to NGOs/institutions operating LPS platforms to support data integration, research capacity building, and community engagement, funding agencies can maximize the impact of their investments. Together, we can foster innovation, drive evidence-based policies, and create sustainable solutions to improve population health and well-being in Africa. By working together, we can transform public health in Africa, build stronger health systems, and create healthier and more resilient communities.

## Supplementary Material

qxae010_Supplementary_Data
